# Perceived neighborhood ethnic diversity and social outcomes: Context-dependent effects within a postindustrial city undergoing regeneration

**DOI:** 10.1080/07352166.2017.1343632

**Published:** 2017-08-16

**Authors:** Ade Kearns, Elise Whitley

**Affiliations:** ^a^ University of Glasgow

## Abstract

This article examines whether perceived neighborhood ethnic diversity is associated with a range of social outcomes in a postindustrial city undergoing regeneration. The research included a survey in 3 types of deprived area in Glasgow: those undergoing regeneration, those directly adjoining regeneration areas, and those further removed from regeneration areas. In areas undergoing regeneration, perceived diversity was positively associated with many residential, cohesion, safety, and empowerment outcomes. This was also true, although to a lesser extent, in deprived areas at some distance from regeneration areas. In areas immediately surrounding the regeneration areas, perceived diversity had mixed associations with residential and safety outcomes and few associations with cohesion and empowerment outcomes. The results suggest that the effects of perceived diversity are context dependent *within* a city. Moreover, regeneration processes alter neighborhood contexts and therefore enable scale, timing, and duration of diversity to mediate the relationships between perceived diversity and social outcomes.

Many cities in Western societies such as the UK are currently experiencing rapid social change as a result of migration and growing ethnic minority populations (Eurostat, 2016). As a result, cities are becoming more ethnically diverse, and some neighborhoods within cities are becoming very multicultural. These changes are seen by people as either positive or negative, depending on factors such as their own recent and past experiences, their general political outlook, and their relative socioeconomic position in relation to migrants (Markaki & Longhi, 2012; Quillian, 1995).

The reality and perceptions of diversity are particularly important to the fortunes of disadvantaged neighborhoods. This is because these neighborhoods experience many of the problems that growing diversity has been shown to either alleviate (e.g., population decline and lack of vitality) or exacerbate (e.g., pressures on often reduced social and public services and amenities; Carley, Campbell, Kearns, Wood, & Young, 2000). In the United States in particular this has led to the so-called racial proxy theory (Harris, 2001; Krysan, 2002; Taub, Taylor, & Dunham, 1984), namely, a perception that people associate neighborhood problems with areas of high Black and ethnic minority residents, whereas, in reality, the root cause of those problems is often lower socioeconomic status. In many countries, such as the UK, it is also the case that migration and ethnic diversity affect disadvantaged neighborhoods more readily and more extensively than they do more affluent neighborhoods (Jivraj & Khan, 2013), often because cheap and available accommodation is present in the former rather than the latter and rapid and unexpected change through increased diversity has been identified as an especially difficult issue for poor communities and those with little prior experience of migration (Hickman, Crowley, & Mai, 2008; Robinson & Walshaw, 2012). The importance of both perceptions and reality is illustrated by survey evidence for the UK, which shows that concern about immigration is generally higher in areas with the lowest numbers of immigrants. The exception to this is that areas where asylum seekers are settled show the highest levels of concern (Duffy, 2014), and these areas are more likely to be disadvantaged neighborhoods. More broadly, the “material context” and “struggles over resources” in disadvantaged neighborhoods are potentially more likely to result in tensions and conflicts between new migrants and existing residents, even if this exists alongside convivial relations (Kesten, Cochrane, Mohan, & Neal, 2011; Robinson & Walshaw, 2012).

This article aims to explore the important relationships between perceptions of ethnic diversity and regeneration in a postindustrial city. Glasgow is undergoing state-sponsored regeneration at the same time as it is experiencing a reversal of population decline and rapid ethnic diversification due to migration. This makes it an ideal setting in which to explore the interplay between the two processes and therefore inform the future study and evaluation of regeneration programs. We consider how perceived diversity forms an important part of the neighborhood contexts that are subject to regeneration treatment and also how changes in perceived diversity can be a by-product of regeneration, which in turn changes the context of both regenerated and other neighborhoods. We examine how perceptions of ethnic diversity vary across three different types of regeneration area within the city: areas of regeneration and redevelopment; areas receiving people relocated from the regeneration areas; and more residentially stable deprived areas receiving housing improvements and additions. By looking at the associations between perceptions of diversity and a number of residential and community outcomes for residents in the three types of area, we may enhance our understanding of regeneration in two respects: firstly, whether the achievement of psychosocial and social objectives^1^ directly through regeneration is either undermined or boosted by perceptions of diversity and, secondly, whether regeneration is indirectly affecting these outcomes through its impacts on perceptions of diversity in core and nearby locations as a result of housing and relocation processes. With respect to diversity, the article addresses the issue of whether the effects of perceived ethnic diversity are context dependent and influenced by the scale, timing, and duration of that diversity, as influenced by regeneration.

## Ethnic diversity and social outcomes

Following Putnam’s (2007) claim that “diversity produces hunkering down” (p. 157), much European research, the majority using objective measures of ethnic diversity (see below), has tested the assumption that where people are visibly and culturally different to one another, they are less likely to socially interact and more likely to be wary of one another due to unfamiliarity or prejudice. Less often studied are the effects of perceptions of diversity. However, there are several reasons why perceptions may be as important, if not more, than objective levels of diversity, not least the racial proxy theory referred to earlier, although its continued sway over and above the effects of actual levels of ethnic residential concentration has recently been questioned in a European context (Dekker, 2013). First, people often overestimate the level of diversity in society or their locality, which serves to exacerbate any concerns they might have. Compared with other European and North American countries, “people in Britain are more likely … to see immigration as a problem rather than an opportunity, and to view the immigrant population as already too large” (Blinder, 2015, p. 6). In addition, survey evidence from the UK has shown that the public overestimates the number of family migrants and asylum seekers in the UK, the latter by a factor of between three and ten (Duffy & Freere-Smith, 2014).

Second, it has been argued that “perceived ethnic density relates to a lower level of geography which is more relevant” for identifying its effects upon health and well-being than the statistical units for which objective measures are available in the UK (Stafford, Becares, & Nazroo, 2009). Perceived ethnic density is said to better reflect co-ethnic presence in people’s “lived neighborhoods,” as well as not relying upon predefined categories of ethnic grouping, which may not correspond to people’s perceptions of who is similar to or different from themselves. Kouvo and Lockmer (2013) also contend that perceived ethnic diversity has advantages over actual ethnic density because “the measure itself contains a subjective evaluation not only of the proportion of ethnic groups, but also the feelings of ethnic distances” and that these related to the “theoretical foundations from intergroup contact theory as well as conflict theory” (p. 3309). Thus, perceptions of diversity at a local level have the potential to be associated with some of the social and psychosocial outcomes sought by regeneration and that form part of the wider social determinants of health and well-being (Dahlgren & Whitehead, 1991). These include neighborhood satisfaction; community cohesion, including social contacts and trust; safety, including antisocial behavior and informal social control; and community empowerment.

Neighborhood satisfaction and enjoyment is considered important in situations where regeneration programs aim to make places more sustainable. The emotional bond between people and place also forms part of personal identity and is said to meet psychological needs and support self-esteem and health and well-being (Guiliani, 2003; Twigger-Ross & Uzzell, 1996; Williams, Patterson, Roggenbuck, & Watson, 1992). Studies across European cities have found a negative association between ethnic diversity (using the Herfindahl index) and neighborhood attachment for indigenous residents (Gorny & Torunczek-Ruiz, 2014), and a study across Dutch towns and cities found that ethnic density was associated with the desire to move (Havekes, Coenders, & van der Lippe, 2014). Consistent with these findings on objective diversity, in one of the few studies of its type, analysis of UK survey data found that high levels of perceived diversity (the respondent’s estimated share of ethnic minorities in the neighborhood) also served to lower people’s neighborhood enjoyment (Uslaner, 2010).

Community cohesion in the form of feeling connected to, having social contacts with, and trusting other people living in the same locality has been a core aim of urban policy and regeneration strategies in the UK since the late 1990s (Kearns, 2003). Several studies have found a negative relationship between neighborhood ethnic diversity (measured using a Herfindahl index or percentage of ethnic minorities) and social contacts (Gijsberts, van der Meer, & Dagevos, 2011; Lancee & Dronkers, 2011; Tolsma, van der Meer, & Gesthuizen, 2009; Vervoort, Flap, & Dagevos, 2011). Negative effects of diversity upon trust have also been reported, albeit in a qualified manner, with lower trust in neighbors but not interethnically (Lancee & Dronkers, 2011), lower trust among the majority group but not other ethnic groups (Gundelach & Freitag, 2014), or trust dependent on the proportion of the next lower-placed ethnic group (in status terms) in the neighborhood (Bakker & Dekker 2012). In European studies, a higher level of perceived neighborhood ethnic mix is associated with more interethnic friendships and lower perceived ethnic threat (Kouvo & Lockmer, 2013; Semyonov & Glikman, 2009). However, perceived ethnic density (measured at the national level) has been associated with higher levels of anti-immigrant sentiment, specifically whether people thought immigrants were good or bad for the economy, culture, and society (Hooghe & de Vroome, 2015). In UK studies, negative associations between ethnic diversity (objectively measured using a Herfindahl index of Theil entropy score) and cohesion/trust have either been absent (Letki, 2008), weak (Twigg, Taylor, & Mohan, 2010), or attenuated by neighborhood compositional and deprivation factors (Becares, Stafford, Laurence, & Nazroo, 2011; Sturgis, Brunton-Smith, Read, & Alum, 2010). In the only study of the effects of perceived diversity upon trust in the UK, Uslaner (2010) reported that generalized trust was higher for all ethnic groups where people live in “integrated neighborhoods” (the perceived share of people from different backgrounds living in the neighborhood being half or more) and have diverse friendship networks (p. 426).

Feeling safe is also an important influence on resident well-being (Kearns, Whitley, Bond, & Tannahill, 2012) and is the first outcome identified as supporting the goal of creating “socially sustainable communities” (Scottish Government, 2011, p. 39). In the United States, fear of crime and perceived risk of victimization have been associated with the perceived ethnic density of the neighborhood (Chiricos, McEntire, & Gertz, 2001). In Australia, overestimated perceived diversity (as measured by the respondent’s estimation of the percentage of non-White residents in the neighborhood) has been associated with higher reported neighborhood disorder (Wickes, Hipp, Zahnow, & Mazerolle, 2013). In the UK, neighborhood ethnic diversity (measured using a Theil entropy score) has been associated with reduced levels of collective efficacy (perceived likelihood of neighbor intervention in local disturbances), although the effects of perceived diversity have not been studied.

Finally, involving the community has become a particular focus of neighborhood renewal policy in the UK since the late 1990s (Imrie & Raco, 2003) and, although ethnic diversity is often equated with dynamism and creativity (McLeod, Lobel, & Cox, 1996), there is no evidence available on the association between diversity and empowerment in a community setting. The current Scottish Regeneration Strategy highlights community empowerment as its primary outcome, with benefits in terms of creativity, effectiveness, and democracy (Scottish Government, 2011). However, Checkoway (2011) has argued that community development, as a strategy to create change, tends to be unitary and needs to be “reconceived as a multicultural process” (p. ii9) that increases communication between groups; that is, diversity can aid community empowerment, given the right approach and supports. However, the effects of perceived diversity upon community empowerment have not been studied previously.

## Regeneration and ethnic diversity

Regeneration aims to improve a range of outcomes for people and places. These can be considered as including residential outcomes (such as neighborhood satisfaction and area reputation), social outcomes (such as interaction and trust, safety, and empowerment), plus enhancements of human and economic capital and the physical environment (Kearns, Tannahill, & Bond, 2009; Lawless & Kearns, 2010). However, both objective and perceived ethnic diversity have been shown to bear associations with some of the key residential and social outcomes sought through regeneration (e.g., Havekes, Coenders, Dekker, & van der Lippe, 2014; Uslaner, 2010; Wickes et al., 2013). This would suggest that if regeneration is enacted in ethnically diverse neighborhoods, the challenge for policymakers and practitioners to achieve their desired outcomes might be harder. Research also suggests that high area deprivation, as seen in regeneration areas, either reverses positive effects of diversity or adds to negative effects, thus complicating the task for regeneration.

In the context of many UK towns and cities where growing and substantial diversity is a relatively recent phenomenon, regeneration programs interact with, and may stimulate, different perceptions of diversity in different locations. In core areas of regeneration activity, diversity through new migration often grows in areas where there is competition for scarce resources, such as decent housing, and therefore “newcomers” are often resented (Esses, Jackson, & Armstrong, 1998). Yet, it is possible that perceptions of newcomers may change over time if migrants are seen as a positive addition to an area that was previously in demographic and social decline. In areas surrounding regeneration sites, changing residential composition as a result of regeneration-related relocation may result in negative perceptions of “incomers,” particularly if those incomers are ethnically diverse in a way the areas were not previously. In this context, communities are not receiving regeneration despite also being deprived and are also having to accommodate incomers who are visibly different in significant numbers, resulting in a negative scenario of imposed, rapid ethnic change (Livingston, Bailey, & Kearns, 2010). In other deprived areas, further out from city centers, incremental improvements and additions to the housing supply may offer residential opportunities to relocated inner-city residents and others, including suburbanizing ethnic minorities (McGarrigle & Kearns, 2009). Though growing ethnic diversity in these traditional White working-class locations may also be perceived negatively, this may be to a lesser degree than in the inner city due to its occurrence at a slower pace and in smaller numbers.

## Regeneration and diversity in Glasgow

The interconnections between diversity and regeneration are very pertinent in the case of Glasgow. Glasgow is one of the most deprived cities in the UK, although the situation is improving: in 2012, 42% of the city’s 694 data zones fell within the 15 most deprived neighborhoods in Scotland, but this was a decrease from the 54% a decade earlier. After several decades of decline, the city’s population has risen to 600,000, regaining half the losses of the previous decade (National Records of Scotland, 2013). One of the main drivers of the population increase has been migration, mainly composed of European migrant workers, economic migrants from Asia, and asylum seekers from Africa and the Middle East (Freeke, 2015). The city’s ethnic minority population has traditionally been small but more than doubled from 42,000 (7.2%) in 2001 to 92,000 (15.4%) in 2011 (Freeke, 2013). Of the city’s 56 planning neighborhoods, 5 had an ethnic minority population of 12% or more in 2001, but by 2010 this had risen to 11 (Freeke, 2012).

There are two main reasons why this city with a small ethnic minority population has become more multicultural. First, after the widening of the European Union in 2004 and 2007, migrants from the Central and Eastern European accession states came to Glasgow, as to other UK cities, living in private rented housing. The number of White residents in the city in 2011 who were not British or Irish was more than double the number in 2001 (Freeke, 2013). Second, Glasgow City Council has, since 2000, been receiving asylum seekers under contractual arrangements with the Home Office. It was recorded that 6,000 asylum seekers were present in the city in 2003 and 2,800 in 2010, with the annual rate of arrival dropping to 1,300 in 2008–2009 and again to 700 in 2009–2010 (Freeke, 2012). The latest available figures are that 1,029 asylum applications were made in Scotland in 2012, the majority in Glasgow (www.scottishrefugeecouncil.org.uk). The number of asylum seekers remaining in the city as refugees after receiving leave to remain is unknown but is likely to be 10,000 or more. Ninety percent of asylum seekers were accommodated in just half a dozen low-demand areas, in order to avoid impacting the housing waiting list for locals (Crawford, Beck, Mclean, Walsh, & Tannahill, 2012). These were mostly high-rise estates of poor-quality accommodation and run-down environments.

It is in this context that Glasgow has been undergoing an extensive program of area regeneration that has affected population numbers and movements between areas within the city. In 2003, eight transformational regeneration areas were declared across the city, each involving widespread demolition and redevelopment, plus seven local regeneration areas, which were smaller in scale and where demolition would be less extensive (Glasgow City Council, 2007; Glasgow Housing Association, 2006). Together, these 15 areas had a large presence of high-rise flats, all cleared and mostly demolished by 2015, and contained a population of 35,000 people, equivalent to 6% of the city’s total, many of whom would have to be moved, although some could remain or return. Several of the regeneration areas were also areas where migrants, particularly asylum seekers and refugees, lived. Thus, the clearance and redevelopment of these areas resulted in the relocation of residents, including migrants, into other, often nearby (Kearns & Mason, 2013), neighborhoods, which had little prior experience of diversity.

Thus, Glasgow is a city where the success or failure of regeneration is entwined with the issue of how residents view the fact that the city has become less deprived, though remaining very deprived, and more diverse. Immigration and multiculturalism have become part of the political project of Scottish devolution, with the nationalist administration pursuing a pro-migration strategy (Scottish Executive, 2004). Although people in Scotland are often described as having relatively positive views of immigration (Rolfe & Metcalf, 2009), their enthusiasm has recently been questioned. Bond (2006) reported that Scots were reluctant to see visible minorities as Scottish, and Pehrson, Brown, & Zagefka (2009) found that those who held an ethnic rather than civil conception of nationalism were relatively opposed to immigration. Most recently, McCollum, Nowok, and Tindal (2014) showed that disadvantaged groups in Scotland, such as those with few qualifications, “are comparatively unreceptive to migrants and overall levels of opposition to migration may be increasing” (p. 99).

It is also the case that the impacts of asylum seekers and refugees upon receiving, deprived communities (those subject to regeneration) can be great, particularly where change happens quickly and unexpectedly and, moreover, where it is the “overall size of the visible BME [Black minority ethnic] population in Glasgow [that] is increasing significantly” (Scottish Refugee Council, 2010, p. 9). In the early years of the asylum resettlement program in Glasgow, there were a high number of incidents of racial harassment on some estates (Binns, 2002), indicating that community cohesion was an issue. As a result, integration networks were established in key parts of the city to support migrants and aid the development of cohesion (ODS Consulting, 2007; Scottish Government, 2003). It is in this context of the changing nature of the city in ethnic terms, intertwined as it is with a political process and entailing additional support interventions to help with integration, that the influence of perceptions of diversity becomes relevant to the goals of regeneration.

## Research aims

This research aims to contribute to existing literature in several ways, both empirical and theoretical. First, through our first three research objectives (see below), we investigate whether perceived ethnic diversity varies between social groups and impacts upon the residential and social outcomes sought in a regeneration context, in ways that evaluations of regeneration may require to take into account. In doing this, we examine a wider range of outcomes than many previous studies and avoid the use of composite indices and thus add to the sparse evidence base on the effects of perceived diversity. Second, through our fourth research objective (see below), we consider how regeneration may change the contexts in which perceptions of diversity have effects, through comparing core areas of regeneration where social and physical changes are greatest in scale, surrounding areas where more recent change is a side effect of the regeneration process, and other areas subject to slower, smaller scale and incremental change. The notion that the effects of perceptions of diversity can be scale related and context dependent within a city, particularly a postindustrial city undergoing regeneration, is worth establishing as a contribution to our understanding of diversity; most studies have looked at whole nations, or cities within nations, and have rarely examined neighborhoods within cities. Lastly, again through our fourth research objective, we explore the crucial role of time in the effects of perceived diversity, in terms of both the speed of physical and social change that feeds into perceptions and the changing impacts of diversity according to its duration. This is important because past studies of perceived diversity have reported cross-sectional findings without considering the influence of time upon perceived diversity or its effects. The more specific research objectives are described below.

### Patterns of perceived neighborhood diversity

Our first objective is to examine who perceives there to be more (or less) ethnic diversity in their neighborhood. This is not a topic often addressed in the literature, although it is important to understand who notices diversity in a city where rapid change has occurred recently. The evidence suggests that if perceptions are influenced by anti-immigrant sentiment, then those in more disadvantaged positions, such as the less educated or unemployed, would have a heightened awareness of diversity and that older people with a more settled ethnic (Scottish) identity may perceive the presence of minorities more than others (Card, Dustmann, & Preston, 2005).

Our second objective is to look at how perceived ethnic diversity varies between areas with different kinds of regeneration intervention. This helps establish whether perceived ethnicity is a potential confounder or mediator of the impacts of regeneration. In realistic evaluation, it is important to understand how context complicates the achievement of objectives (Blamey & Mackenzie, 2007). In the case of Glasgow, growing diversity is a coincident change taking place in deprived areas subject to regeneration treatments. We expect levels of perceived diversity to be highest in the regeneration areas but also relatively high in their adjacent surrounding areas, while being somewhat lower in other disadvantaged locations. We are using a smaller scale than used in some other studies, which the literature suggests is more appropriate where perceptions of diversity are being investigated. Our study areas are also natural communities rather than statistical units.

### Perceived neighborhood diversity and residential and social outcomes

Our third objective is to establish whether associations exist between perceived diversity and residential, cohesion, safety, and empowerment outcomes in deprived areas in Glasgow. Because past studies report that area deprivation is often the more important driver of negative outcomes, we offer a more robust exploration of the effects of perceived diversity, because all of our study areas are very deprived. We also use individual items within each domain of interest, an advantage over studies that include different indicators, such as for social interaction and trust, within composite scales. We investigate community empowerment as an outcome, which has not been done previously. On the basis of the literature reviewed, we would expect to find negative associations between perceived diversity and residential, social, safety, and empowerment outcomes, due to a mixture of factors including unfamiliarity with newcomers, aversion to social change, tensions, conflicts and competition between settled and migrant groups, and perceived lack of control over community change (Bakker & Dekker, 2012; Chiricos et al., 2001; Havekes, Coenders, Dekker, & van der Lippe, 2014; Livingston et al., 2010).

Our final objective is to see whether any associations between perceived diversity and the various outcomes vary between the three types of area: regeneration areas, surrounding areas, and other areas. We expect to observe some differences between regeneration areas and surrounding areas, due to different recent histories of diversity and as a result of the regeneration process. The three-way spatial comparison also enables us to consider the role of time in the effects of perceived diversity because regeneration areas underwent rapid compositional change some time ago and have experienced diversity over a longer period of time, whereas the surrounding areas have undergone rapid compositional change more recently and the other areas have experienced slow compositional change over a longer period of time.

In regeneration areas, we might expect perceived diversity to be positively (rather than negatively) associated with social outcomes due to the influx of a new social dynamic into areas that have lacked social investment and have been declining through losing population. Social activity in such areas may be seen to have increased both due to host community assistance to migrants and due to migrant self-help activity (Stewart & Shaffer, 2015). A negative association with feelings of safety may have dissipated in regeneration areas over time as tensions have eased (Kearns & Whitley, 2015) but may still exist in surrounding areas where relocation of migrants is more recent. There may be a positive association between perceived diversity and community empowerment in regeneration areas as a result both of the operation of refugee integration projects in those areas (ODS Consulting, 2007) and due to the efforts to engage the community in the process of regeneration itself (Lawson & Kearns, 2010); these positive factors would not exist in the other two types of area, where lack of control over change may underlie a negative association with empowerment.

## Methods

### Data source

The data come from a face-to-face survey of adults carried out in 15 communities across the city of Glasgow in 2011. One person was interviewed at each address, either the head of household or his or her partner. This was the third wave of a long-term study of the impacts of housing improvements and regeneration on communities and individuals (Egan et al., 2010). The study communities were all locally known and locally defined entities (e.g., a named housing estate) and ranged in size from 1,000 to 10,000 people at the start of the study. They are relatively deprived, all but one falling within the 15% most deprived areas of Scotland, using the government’s measure of income deprivation based on receipt of out-of-work benefits (Walsh, 2008). The study is structured to include five types of intervention area, which we have grouped into three types of areas for the purposes of the current analysis, as follows: (a) regeneration areas, which include three of the transformational regeneration areas and three of the local regeneration areas mentioned above; (b) two wider surrounding areas, which include several contiguous neighborhoods lying adjacent to the regeneration areas and which were expected to receive people relocated out of the high-rise estates; and (c) other areas, which include five areas of housing improvement works and two postwar peripheral housing schemes. In nine study areas (groups b and c), a random sample of addresses was selected, stratified by subareas (32 subdivisions of the communities) and housing tenure (social rented sector, private rented, and owner occupier). In the six regeneration areas undergoing extensive redevelopment (group a), all existing addresses at the time of survey were selected due to uncertainty about which properties were/were not still occupied and declining numbers of dwellings over time. The survey targeted adults aged over 16 who were householders (tenants or owners) or their partners and achieved a 45% response rate, with 4,302 completed interviews. Although we might have expected a lower response rate in areas of regeneration activity, this was not the case, with nonresponse and refusal rates similar between these areas and others. The survey asked respondents for their views about their housing, neighborhoods, community and social contacts, physical and mental health, and health behaviors, mostly using questions from other (often national) surveys but including some questions developed ourselves.

### Measures used

The key independent variable of interest and our measure of perceived diversity was an original question that asked respondents: “How mixed do you think your neighbourhood is in terms of the ethnic background of the residents?” with the response choices being *very mixed, fairly mixed*, and *hardly mixed at all*. Neighborhood was defined for respondents as “the local area within a 5- to 10-minute walk of your home.”

Outcome variables were considered in four groups, covering (a) residential benefits, (b) community cohesion, (c) safety, and (d) empowerment. Variables were based on self-report questions, which had between four and six possible response categories. For ease of interpretation, these were dichotomized in the current analyses, focusing on the most “positive” single response or most positive two responses if the most positive single response accounted for <20% of respondents. The wordings of all of the survey questions and the dichotomization of response categories for analysis are presented in Table 1. For example, neighborhood satisfaction was based on the question, “How satisfied or dissatisfied are you with this neighbourhood as a place to live?” with analysis comparing those who gave the response *very satisfied* versus all other responses combined.

**Table 1.** Outcome variables.

Variable	Question wording	Predicted outcome		
*Residential outcomes*				
Neighborhood satisfaction	“How satisfied or dissatisfied are you with this neighborhood as a place to live?”	Very satisfied	vs.	Fairly satisfied, neither, fairly dissatisfied, very dissatisfied
Neighborhood enjoyment	“I enjoy living here.”	A great deal	vs.	A fair amount, not very much, not at all
Progress from home	“My home makes me feel that I’m doing well in life.”	Strongly agree, agree	vs.	Neither, disagree, disagree strongly
Progress from neighborhood	“Living in this neighborhood helps make me feel that I’m doing well in life.”	Strongly agree, agree	vs.	Neither, disagree, disagree strongly
Internal area reputation	“People who live in this neighborhood think highly of it.”	Strongly agree, agree	vs.	Neither, disagree, disagree strongly
External area reputation	“Many people in Glasgow think this neighborhood has a bad reputation.”	Strongly disagree, disagree	vs.	Neither, agree, strongly agree
*Cohesion outcomes*				
Belonging	“I feel I belong to this neighborhood.”	A great deal	vs.	A fair amount, not very much, not at all
Inclusion	“I feel part of the community.”	A great deal	vs.	A fair amount, not very much, not at all
Neighbor interaction	“I stop and talk to people in my neighborhood.”	A great deal	vs.	A fair amount, not very much, not at all
Neighbor familiarity	“Would you say that you know … people in your neighborhood?”	Most, many	vs.	Some, very few, no one
Neighborly concern	“My neighborhood is a place where neighbors look out for each other.”	A great deal	vs.	A fair amount, not very much, not at all
Neighbor honesty	“Someone who lost a purse or wallet around here would be likely to have it returned without anything missing.”	Strongly agree, agree	vs.	Neither, disagree, disagree strongly
*Safety outcomes*				
Safety at night	“How safe would you feel walking alone in this neighborhood after dark?”	Very safe, fairly safe	vs.	Neither, a bit unsafe, very unsafe, never walk alone after dark
Informal social control	“It is likely that someone would intervene if a group of youths were harassing someone in the local area.”	Strongly agree, agree	vs.	Neither, disagree, disagree strongly
Antisocial behavior	“Could you tell me whether each of the following things is a serious problem, a slight problem, or not a problem in your local neighborhood: vandalism; violence, including assaults; intimidation; racial harassment; drug use or dealing; drunk or rowdy behavior; gang activity; teenagers hanging around; nuisance neighbors; burglary.”	None	vs.	One or more serious problems
*Empowerment outcomes*				
Influence	“On your own, or with others, you can influence decisions affecting your local area.”	Strongly agree, agree	vs.	Neither, disagree, disagree strongly
Proactivity	“People in this area are able to find ways to improve things around here when they want to.”	Strongly agree, agree	vs.	Neither, disagree, disagree strongly
Responsiveness	“The providers of local services, like the council and others, respond to the views of local people.”	Strongly agree, agree	vs.	Neither, disagree, disagree strongly

For *residential benefits*, we first focused on measures of neighborhood satisfaction and enjoyment, which have featured in past studies of ethnic diversity (e.g., Uslaner, 2010). Given the link between ethnic minority residence and area deprivation in the UK (Jivraj & Khan, 2013), we also considered status-related psychosocial benefits (sense of progress) from the home and neighborhood as potential outcomes from perceived diversity, as well as the perceived internal and external reputation of their neighborhood; that is whether respondents agreed that local people “thought highly” of the area and/or that people elsewhere thought the area had a “bad reputation.” These psychosocial variables have been shown in the past to bear strong associations with mental well-being (Kearns et al., 2012).

Other UK research has shown that place attachment falls for White residents at higher levels of (objectively measured) ethnic diversity (Bailey, Kearns, & Livingston, 2012) and, thus, for *community cohesion* outcomes we examined associations of perceived diversity with belonging, social engagement, and trust. For belonging, respondents were asked how much they felt that they belonged to the neighborhood and how much they felt part of the community. For social engagement, respondents were asked to what extent they stopped and talked to people in the neighborhood and how many people in the neighborhood they knew. In relation to trust, respondents were asked firstly about reliance on others—that is, whether their neighbors “looked out for each other”—and, secondly, about the honesty of their neighbors; that is, whether they thought it was likely that a lost purse or wallet would be returned intact. These community cohesion measures have been used by the UK government since 2001 in its Citizenship Survey to monitor the promotion of equality and community cohesion (Kitchen, Michaelson, & Wood, 2006).

Because it has been shown the people’s understanding of the ethnic diversity of their neighborhoods influences the level of their concern about social disorder (Sampson, 2009), we consider aspects of *safety* in relation to perceived diversity. Respondents were asked about the likelihood of neighborly informal social control, how safe they would feel walking alone in the neighborhood after dark, and which of a list of 10 antisocial behavior issues (ranging from crime to disturbance and nuisance) they considered to be a serious problem in their neighborhood, something that pertains both to safety and shared norms in the area. The question on safety is used to monitor public fear of crime in the Scottish Crime and Justice Survey (Scottish Government, 2016) and those on antisocial behavior are used to monitor neighborhood conditions in the Scottish Household Survey (Scottish Household Survey Project Team, 2016).

With regard to local empowerment, it has been shown that non-Whites in the UK are less likely to act together as members of associational groups than Whites (Warde et al., 2003), although, conversely, Black and Asian people are both more likely to feel a degree of influence over local decisions (Kitchen, Michaelson, Wood, & John, 2006) and to be more trustful of regeneration partnerships (Grimsley, Hickman, Lawless, Manning, & Wilson, 2005). These ethnic differences raise the question of whether perceived neighborhood diversity influences residents’ feelings of empowerment. Respondents were therefore asked about three kinds of collective *empowerment*, termed influence (whether alone or with others they could influence decisions affecting their area), proactivity (whether the community could find its own ways to improve things when they wanted to), and responsiveness (whether public service providers were responsive to things the community said). The first of these questions is a national indicator of public service performance in Scotland and is also widely used in community surveys (Scottish Government, 2015); the latter two items are original questions developed for this study.

### Analysis

Logistic regression was used to explore the associations of the dichotomized outcome variables with perceived ethnic diversity. The analyses were conducted separately for the three intervention area types stated above, namely, regeneration areas, surrounding areas, and other areas. Odds ratios (ORs) and associated 95% confidence intervals (CIs) for the positive outcomes are presented using the hardly mixed group as the reference category. ORs are adjusted for a number of individual-level confounding variables likely to affect the outcomes and perceived ethnic diversity, including age group (16–39, 40–64, 65+), sex, household type (adult, single-parent family, two-parent family, older person), employment status (employed, unemployed, long-term sick, home keeper, retired), ethnicity (British, non-British), and years living in the area (5 or less, 6–20, 21 or more). Confidence intervals are based on robust standard errors, allowing for clustering within study areas. Analyses were based on respondents with complete data for perceived ethnic diversity, confounders, and the outcome of interest. Our analyses have resulted in a large number of comparisons and, on occasion, are based on relatively small numbers of respondents, particularly in the case of analyses stratified by area type, and there is therefore potential for both type I and type II errors. Our interpretation of these results therefore focuses on broad, consistent patterns rather than limiting attention to isolated results that are conventionally statistically significant.

## Results

### Patterns of perceived neighborhood diversity

The original survey included 4,302 respondents. In total 444 (10.3%) had missing values for perceived ethnic diversity or confounding variables, leaving 3,858 (89.7%) in our analytical sample (Table 2). Respondents excluded from the analyses were not markedly different from those included. Among those included in the analyses, 1,342 (34.8%) of respondents were drawn from regeneration areas, 769 (19.9%) from surrounding areas, and 1,747 (45.3%) from other areas. Respondents in regeneration areas were generally younger, were more likely to be unemployed, were less likely to be British, and had generally lived in the area for a shorter period than those in surrounding or other areas. Perceptions of ethnic diversity were highest in the regeneration areas, as expected, with two out of five residents considering their neighborhood to be very mixed in ethnic terms. Perceptions of diversity were very similar overall in the other two types of areas, with far fewer residents considering their neighborhood to be very mixed: one in nine people in the surrounding areas and one in seven in the other areas. Comparing these responses with published spatial data on the non-White population across the city, we see a broad correspondence between perceptions and reality: the ethnic density in regeneration areas is approximately three times that of the other two types of areas and, accordingly, perceptions of neighborhoods being very mixed were three times as prevalent in the regeneration areas as elsewhere.

**Table 2.** Perceived ethnic diversity by area type (column percentage).

	Regeneration areas *N* = 1,342	Surrounding areas *N* = 769	Other areas *N* = 1,747
Sex			
Male	44.3	39.8	39.4
Female	55.7	60.2	60.6
Age			
<40	50.1	25.5	27.1
40–64	35.8	47.5	46.2
65+	14.1	27.1	26.7
Household type			
Adult	46.9	42.4	44.7
Single parent	20.6	13.8	14.6
Two parent	17.9	15.9	12.7
Older	14.5	28.0	28.0
Employment status			
Employed	26.8	36.0	25.8
Unemployed	34.3	15.0	14.7
Long-term sick	11.5	10.5	17.3
Home keeper	11.8	6.8	9.7
Retired	15.7	31.7	32.4
Citizenship			
British	65.5	97.1	93.7
Non-British	34.5	2.9	6.3
Years in area			
5 or less	47.8	18.2	25.1
6–20	29.4	28.9	24.3
21 or more	22.7	52.9	50.7
Perceived ethnic diversity			
Hardly mixed at all	10.4	38.5	40.1
Fairly mixed	45.3	50.1	44.8
Very mixed	44.3	11.4	15.1
Mean ethnic diversity^a^	38.3	14.7	12.9

*Note*. ^a^Mean percentage of residents non-White British/Irish, across relevant data zones.

Source: Data from UK Census 2011.

Table 3 presents perceptions of neighborhood ethnic diversity by the sociodemographic measures used in the study. Results are presented separately for the three area types to account for differences in sociodemographic characteristics of respondents. Within each area type, there was little indication that men and women have different perceptions of ethnic diversity in their neighborhood. However, older people (aged 65+), older households, and retirees, particularly in surrounding areas, were much more likely than other respondents to consider that they live in neighborhoods that are hardly mixed in ethnic terms. Conversely, parents and those who had lived in the neighborhoods the least time (5 years or less) were much more likely than others to consider that their neighborhood is fairly or very mixed in ethnic terms. Non-British respondents, particularly in surrounding and other areas, were more likely than British respondents to perceive high ethnic neighborhood mix.

**Table 3.** Perceived ethnic diversity by sociodemographic factors (row percentages).

	% of respondents rating ethnic diversity of area as hardly/fairly/very mixed		
	Regeneration areas *N* = 1,342	Surrounding areas *N* = 769	Other areas *N* = 1,747
Sex			
Male	9.4/44.6/46.0	36.9/52.6/10.5	38.2/44.7/17.1
Female	11.1/45.9/43.1	39.5/48.4/12.1	41.4/44.9/13.7
Age			
<40	8.2/44.8/47.0	23.0/63.8/13.3	32.8/49.1/18.2
40–64	10.8/45.1/44.1	40.0/47.7/12.3	42.3/43.5/14.3
65+	16.9/47.6/38.5	50.5/41.4/8.2	43.9/42.8/13.3
Household type			
Adult	10.2/45.6/44.3	36.8/50.6/12.6	41.5/43.0/15.5
Single parent	7.6/43.7/48.7	31.1/54.7/14.2	33.3/50.2/16.5
Two parent	9.2/44.6/46.3	25.4/61.5/13.1	36.5/47.8/15.8
Older	16.4/47.7/35.9	52.1/40.5/7.4	43.2/43.6/13.3
Employment status			
Employed	6.7/46.4/46.9	31.4/54.2/14.4	38.4/45.0/16.6
Unemployed	8.5/43.5/48.0	31.3/57.4/11.3	35.4/48.6/16.0
Long-term sick	12.3/44.2/43.5	39.5/46.9/13.6	42.2/44.9/12.9
Home keeper	14.6/48.1/37.3	23.1/63.5/13.5	35.9/47.1/17.1
Retired	16.2/46.2/37.6	52.9/40.2/7.0	43.8/42.2/14.0
Citizenship			
British	12.0/46.1/42.0	39.4/48.9/11.8	41.5/44.4/14.1
Non-British	7.3/43.8/48.8	0.0/9.1/90.9	20.0/50.9/29.1
Years in area			
5 or less	8.4/43.5/48.1	30.7/57.9/11.4	35.2/45.9/19.0
6–20	12.2/46.1/41.8	31.1/55.9/13.1	38.0/44.6/17.5
21 or more	12.1/48.2/39.7	45.2/44.2/10.6	43.6/44.4/12.0

### Perceived neighborhood diversity and residential and social outcomes

Associations of perceived ethnic diversity and residential outcomes are presented by area type in Table 4. There was no consistent association between perceived ethnic density and neighborhood satisfaction among respondents in regeneration areas. However, respondents in surrounding areas who reported greater ethnic mix were less likely to report being satisfied with their neighborhood (OR for very mixed = 0.51, 95% CI, 0.27, 0.96), whereas those in other areas who considered their neighborhood to be very mixed were markedly more likely to report satisfaction (OR = 1.57, 95% CI, 1.12, 2.21). Respondents in regeneration areas who considered their neighborhood to be more ethnically diverse were more likely to agree that they enjoyed living there (OR for very mixed = 1.59, 95% CI, 1.08, 2.34); conversely, those living in surrounding areas considered to be fairly mixed were less likely to agree that they enjoyed living there (OR = 0.69, 95% CI, 0.51, 0.92). Respondents who considered their neighborhoods to be fairly mixed (regeneration and other areas) or very mixed (surrounding and other areas) were more likely to agree that their home made them feel they were doing well in life, although 95% CIs did not always exclude 1. There was little evidence of any association between perceived ethnic density and feelings of progress from the neighborhood or perceptions of the internal reputation of the area. However respondents in the other areas who considered their neighborhood to be very mixed were less likely to disagree that the area had a bad external reputation (OR = 0.58, 95% CI, 0.37, 0.90) compared to those who considered their neighborhood to be hardly mixed.

**Table 4.** Residential outcomes according to perceived ethnic density: Odds ratios for better outcomes^a^ (95% confidence intervals).^b^

	Regeneration areas (*N* = 1,342)	Surrounding areas (*N* = 769)	Other areas (*N* = 1,747)
Neighborhood satisfaction			
Hardly mixed	1.00	1.00	1.00
Fairly mixed	0.73 (0.42, 1.28)	0.61 (0.42, 0.88)	0.93 (0.76, 1.13)
Very mixed	0.96 (0.53, 1.76)	0.51 (0.27, 0.96)	1.57 (1.12, 2.21)
Neighborhood enjoyment			
Hardly mixed	1.00	1.00	1.00
Fairly mixed	1.26 (0.62, 2.58)	0.69 (0.51, 0.92)	0.83 (0.58, 1.18)
Very mixed	1.59 (1.08, 2.34)	1.15 (0.81, 1.65)	1.27 (0.88, 1.85)
Progress from home			
Hardly mixed	1.00	1.00	1.00
Fairly mixed	1.55 (1.06, 2.28)	1.05 (0.76, 1.44)	1.38 (1.02, 1.88)
Very mixed	0.99 (0.70, 1.41)	1.34 (0.84, 2.13)	1.31 (0.95, 1.79)
Progress from neighborhood			
Hardly mixed	1.00	1.00	1.00
Fairly mixed	1.12 (0.64, 1.97)	1.12 (0.70, 1.79)	1.06 (0.79, 1.43)
Very mixed	1.00 (0.70, 1.44)	1.03 (0.54, 1.96)	0.87 (0.59, 1.29)
Internal reputation			
Hardly mixed	1.00	1.00	1.00
Fairly mixed	1.06 (0.50, 2.24)	0.99 (0.88, 1.12)	1.14 (0.81, 1.61)
Very mixed	0.88 (0.43, 1.80)	0.90 (0.53, 1.53)	1.16 (0.90, 1.50)
External reputation			
Hardly mixed	1.00	1.00	1.00
Fairly mixed	0.73 (0.45, 1.18)	0.91 (0.79, 1.04)	0.87 (0.58, 1.31)
Very mixed	0.72 (0.47, 1.10)	1.09 (0.49, 2.42)	0.58 (0.37, 0.90)

*Note*. ^a^Adjusted for age group, sex, household type, employment status, citizenship, and years in area.

^b^95% confidence intervals based on robust standard errors accounting for clustering within area.

Among respondents in regeneration areas, those perceiving their neighborhood to be more ethnically diverse were more likely to agree with positive cohesion outcomes (Table 5; e.g., OR for belonging = 1.46, 95% CI, 1.11, 1.91; OR for inclusion = 1.85, 95% CI, 1.23, 2.79; OR for neighborhood interaction = 2.21, 95% CI, 1.74–2.82; and OR for neighborly concern = 1.74, 95% CI, 1.01,2.99), although 95% CIs for fairly mixed generally included 1 and, in the case of neighbor familiarity and neighbor honesty, respondents in areas perceived to be fairly mixed were more likely to agree with the outcome statement than those who perceived their area to be very mixed. Similar positive associations were observed in other areas, particularly for neighbor familiarity (OR = 1.38, 95% CI, 1.01–1.87), neighborly concern (OR = 1.36, 95% CI, 0.89–2.08), and neighbor honesty (OR = 1.54, 95% CI, 0.98–2.42), although these were generally weaker than those observed in regeneration areas. There were no strong associations with ethnic diversity and cohesion outcomes in surrounding areas, with the exception of stronger feelings of belonging where neighborhoods were perceived to be very mixed (OR = 1.49, 95% CI, 1.24–1.80).

**Table 5.** Cohesion outcomes according to perceived ethnic density: Odds ratios for better outcomes^a^ (95% confidence intervals).^b^

	Regeneration areas (*N* = 1,342)	Surrounding areas (*N* = 769)	Other areas (*N* = 1,747)
Belonging			
Hardly mixed	1.00	1.00	1.00
Fairly mixed	1.07 (0.63, 1.81)	0.84 (0.61, 1.14)	0.84 (0.49, 1.43)
Very mixed	1.46 (1.11, 1.91)	1.49 (1.24, 1.80)	1.22 (0.70, 2.11)
Inclusion			
Hardly mixed	1.00	1.00	1.00
Fairly mixed	1.32 (0.82, 2.12)	0.72 (0.62, 0.82)	0.92 (0.55, 1.54)
Very mixed	1.85 (1.23, 2.79)	0.96 (0.87, 1.07)	1.26 (0.72, 2.19)
Neighborhood interaction			
Hardly mixed	1.00	1.00	1.00
Fairly mixed	1.64 (0.91, 2.97)	0.89 (0.63, 1.26)	0.94 (0.66, 1.34)
Very mixed	2.21 (1.74, 2.82)	1.17 (0.69, 1.97)	1.27 (0.81, 1.99)
Neighbor familiarity			
Hardly mixed	1.00	1.00	1.00
Fairly mixed	1.59 (1.01, 2.49)	0.97 (0.72, 1.29)	1.28 (1.03, 1.60)
Very mixed	1.29 (0.81, 2.03)	1.22 (0.60, 2.45)	1.38 (1.01, 1.87)
Neighborly concern			
Hardly mixed	1.00	1.00	1.00
Fairly mixed	1.50 (0.73, 3.09)	0.90 (0.70, 1.16)	1.15 (0.99, 1.34)
Very mixed	1.74 (1.01, 2.99)	1.01 (0.67, 1.51)	1.36 (0.89, 2.08)
Neighbor honesty			
Hardly mixed	1.00	1.00	1.00
Fairly mixed	1.65 (0.62, 4.39)	1.01 (0.81, 1.26)	1.36 (0.98, 1.88)
Very mixed	1.10 (0.48, 2.52)	0.93 (0.77, 1.12)	1.54 (0.98, 2.42)

*Note*. ^a^Adjusted for age group, sex, household type, employment status, citizenship, and years in area.

^b^95% confidence intervals based on robust standard errors accounting for clustering within area.

Respondents in all areas who perceived their neighborhood to be more ethnically diverse were more likely to agree that they felt safe at night (Table 6), particularly those living in surrounding areas (OR for very mixed = 1.98, 95% CI, 0.57–6.90). Those living in regeneration areas and who considered their neighborhood to be ethnically diverse were also more likely to agree with the statement regarding informal social control and to report no serious antisocial behavior problems, although associations were stronger among those who said that the neighborhood was fairly mixed (OR for informal social control = 1.83, 95% CI, 0.91, 3.68; OR for no antisocial behavior problems = 1.48, 95% CI, 1.00, 2.20) rather than very mixed. In contrast, there were no strong associations between perceived ethnic diversity and informal social control or antisocial behavior in other areas and suggestions of negative associations among respondents in surrounding areas who considered their neighborhood to be very mixed, although 95% CIs did not exclude 1 (OR for informal social control = 0.66, 95% CI, 0.42, 0.04; OR for antisocial behavior = 0.77, 95% CI, 0.48, 1.23).

**Table 6.** Safety outcomes according to perceived ethnic density: Odds ratios for better outcomes^a^ (95% confidence intervals).^b^

	Regeneration areas (*N* = 1,342)	Surrounding areas (*N* = 769)	Other areas (*N* = 1,747)
Safety at night			
Hardly mixed	1.00	1.00	1.00
Fairly mixed	1.42 (1.02, 1.98)	1.23 (0.93, 1.64)	1.18 (1.03, 1.36)
Very mixed	1.17 (1.00, 1.37)	1.98 (0.57, 6.90)	1.39 (1.02, 1.90)
Informal social control			
Hardly mixed	1.00	1.00	1.00
Fairly mixed	2.42 (0.97, 6.06)	1.00 (0.67, 1.51)	1.20 (0.70, 2.04)
Very mixed	1.83 (0.91, 3.68)	0.66 (0.42, 1.04)	1.08 (0.83, 1.59)
Antisocial behavior			
Hardly mixed	1.00	1.00	1.00
Fairly mixed	2.00 (1.42, 2.83)	0.79 (0.70, 0.89)	1.00 (0.87, 1.14)
Very mixed	1.48 (1.00, 2.20)	0.77 (0.48, 1.23)	0.85 (0.56, 1.28)

*Note*. ^a^Adjusted for age group, sex, household type, employment status, citizenship, and years in area.

^b^95% confidence intervals based on robust standard errors accounting for clustering within area.

There were strong positive associations between perceived ethnic diversity and all empowerment outcomes among respondents living in other areas (e.g., OR for influence = 2.05, 95% CI, 1.43, 2.93; Table 7), with ORs for agreement with positive statements increasing consistently with greater perceived diversity. Similar, although weaker, increasingly positive associations with perceived diversity were also observed for agreement with proactivity (OR = 1.59, 95% CI, 0.65, 3.90) and responsiveness (OR = 1.65, 95% CI, 0.71, 3.82) among respondents living in regeneration areas, although 95% CIs did not exclude 1. Associations in surrounding areas were generally null or weak, with a single increased odds of agreement with positive proactivity in those perceiving their area as fairly mixed (OR = 1.59, 95% CI, 1.11, 2.27).

**Table 7.** Empowerment outcomes according to perceived ethnic density: Odds ratios for better outcomes^a^ (95% confidence intervals).^b^

	Regeneration areas (*N* = 1,342)	Surrounding areas (*N* = 769)	Other areas (*N* = 1,747)
Influence			
Hardly mixed	1.00	1.00	1.00
Fairly mixed	1.12 (0.47, 2.63)	1.17 (0.94, 1.46)	1.41 (1.08, 1.84)
Very mixed	0.97 (0.41, 2.25)	1.00 (0.95, 1.05)	2.05 (1.43, 2.93)
Proactivity			
Hardly mixed	1.00	1.00	1.00
Fairly mixed	1.45 (0.55, 3.83)	1.59 (1.11, 2.27)	1.32 (0.84, 2.05)
Very mixed	1.59 (0.65, 3.90)	1.10 (0.62, 1.97)	1.89 (1.03, 3.46)
Responsiveness			
Hardly mixed	1.00	1.00	1.00
Fairly mixed	1.45 (0.59, 3.56)	1.02 (0.82, 1.27)	1.39 (1.07, 1.81)
Very mixed	1.65 (0.71, 3.82)	0.95 (0.36, 2.49)	1.67 (1.04, 2.69)

*Note*. ^a^Adjusted for age group, sex, household type, employment status, citizenship, and years in area.

^b^95% confidence intervals based on robust standard errors accounting for clustering within area.

## Discussion

### Patterns of perceived neighborhood diversity

We have studied neighborhood-level perceived ethnic diversity in a city that has changed its ethnic composition markedly since the turn of the millennium, to explore who, in such circumstances, most notices the growth in ethnic diversity and whether perceptions of diversity vary between areas experiencing different types of regeneration. As expected, we found that perceptions of diversity were highest among those who are unemployed and who may be affected by migrant competition for jobs and among migrants themselves who tend to be concentrated in areas with varied ethnicities and nationalities. Contrary to our expectations, older people had the lowest perceptions of diversity, possibly reflecting the fact that they tend to live in more homogenous, White neighborhoods; we did not ask about perceptions of diversity at the city or national level, where they may have more awareness or concern. In addition, and also as expected, we found perceptions of diversity to be highest in regeneration areas, where migrants tend to be most clustered in the poorest conditions. We found perceptions of diversity to be much lower in both the surrounding areas and other areas, which may indicate that levels of diversity have to be much higher than the city average for people to notice much.

### Perceived ethnic diversity and residential and social outcomes

With regard to residential outcomes, we found several instances where respondents in particular areas who perceived ethnic diversity to be high were more likely to agree with statements regarding positive residential outcomes; for example, neighborhood satisfaction and enjoyment. However, we did not find perceived ethnic diversity to be associated with two residential psychosocial outcomes found to be important for mental well-being in previous analyses: sense of progress from the neighborhood and internal area reputation (Kearns et al., 2012). In regeneration and other areas we also found that respondents who perceived their neighborhood to be more ethnically diverse were less likely to disagree that the area had a bad external reputation, a more negative response, and associations were stronger for those who considered the neighborhood to be very rather than fairly mixed. This may partly reflect spatial inequalities in British cities, whereby ethnic minorities are more likely to live in deprived neighborhoods than the White British population (Jivraj & Khan, 2013), so that residents of those areas may see ethnic diversity as a marker of difference from other, more affluent areas. It has also been suggested that in poor areas, feelings about ethnic minorities in the area might not reflect experiences of that group as much as perceptions of neighborhood conditions and expectations as to how the neighborhood will develop in the future, again something particularly pertinent in deprived areas (Havekes, Coenders, & van der Lippe, 2014).

In the regeneration areas in particular, and contrary to past studies, we found greater agreement with a number of cohesion outcomes (pertaining to belonging, inclusion, interaction, familiarity, and neighborly concern) among those who perceived their neighborhood to be more ethnically diverse; we discuss why this might be the case in the next section. To a lesser extent, this was also the case for a number of outcomes (familiarity, neighborly concern, and honesty) in the other areas, where the overall ethnic minority presence is lower, regardless of perception, and/or where change in neighborhood composition has occurred more slowly in absolute terms (Freeke, 2013). Dutch research has found instances where a larger ethnic minority group in a neighborhood was associated with less positive attitudes toward them and where, for some ethnic groups, it was not the current size of the ethnic minority group but its increasing share of the local population that “promoted” interethnic conflict, competition and threat, and less positive attitudes (Blumer, 1958; Bobo & Hutchings, 1996; Savelkoul, Scheepers, Tolsma, & Hagendoorn, 2010). Similarly, UK research, in deprived areas in particular, has reported that ethnic diversity was viewed positively in areas of greater stability over time and more negatively with detrimental impacts upon place attachment in areas where there had been a rapid increase in the ethnic minority population (Livingston et al., 2010). These findings from The Netherlands and from the UK are consistent with our findings of greater neighborhood trust and reliance alongside higher perceived ethnic diversity in the other areas untouched by rapid recent change.

Although evidence from the criminal justice system for England and Wales indicates that non-Whites are both more likely to be victims of crime and more likely to be convicted and imprisoned for committing crimes (Ministry of Justice, 2013), we found several instances where perceived ethnic diversity was associated with stronger agreement with statements regarding safety and control, especially in regeneration areas, although, in general, the most positive responses were among those who considered their area to be fairly rather than very mixed. This may be partly a reflection of the positive cohesion outcomes reported above for areas of higher perceived diversity but may also be due to the public policy response to the presence of large numbers of asylum seekers and refugees in some Glasgow neighborhoods over the previous decade. This has involved social support and integration projects as well as a stronger police response to initial problems of racial tension and harassment in areas of migrant settlement (ODS Consulting, 2007; Scottish Government, 2003). It may be the case that residents generally have noticed enhancements in guardianship and safety over time in areas of greater diversity.

As we surmised, there were also several instances where we found responses to empowerment outcomes to be more positive among respondents who perceived themselves to be living in areas of higher ethnic diversity, particularly in other areas, unaffected by regeneration. We can identify a number of possible reasons for this, although we cannot determine which is more applicable from our research. On the one hand, it may be that a collective mentality of dependency on official authorities, possibly due to unrealized past ambitions resulting in low self-efficacy and so-called learned helplessness in areas of long-term deprivation (Peterson, Maier, & Seligman, 1995), is disrupted by the presence of ethnic minorities and migrants, leading to an increased sense of self-realization in the host community. Conversely, migrants are often reported to be more educated and aspirational than others (e.g., Czaika & Vothknecht, 2014) and are “frequently described admiringly as ‘hard workers’” (Lewis, 2006, p. 17), and it may be the case that their attitudes and capabilities are observed, admired, or adopted by other residents. Lastly, deprived areas receiving relatively poor service provision (Hastings, 2009) can be altered by the arrival of migrants, with additional services provided both for migrants specifically and to assist with the integration of whole communities (ODS Consulting, 2007), and this greater attention and intensity of service provision for migrants may result in greater feelings of empowerment. It is worth noting, however, that, in regeneration areas in the current analysis, there was no association between perceived diversity and respondents’ influence over key decisions affecting the area, such as major planning and development issues; this is perhaps an area where the authorities’ grip on power is least likely to be loosened by changes in the resident population.

### Regeneration and context influences upon the associations of perceived diversity

Regeneration areas, by virtue of their property types (typically high-rise housing) and experience of low demand for housing, have produced particular residential contexts that have been used for the last 15 years or more for the housing of asylum seekers, refugees, and, to a lesser extent, economic migrants (Crawford et al., 2012). These areas are reported to have experienced, and overcome, a difficult initial period of incorporation of migrants at short notice (FMR Research, 2003). At the time of our survey, these areas had relatively high proportions of ethnic minority residents (around two out of five residents) but also a decade and a half’s experience of such presence. In these areas, we found perceived ethnic diversity to be positively associated with several outcomes pertaining to residential satisfaction, feelings of safety and empowerment, and cohesion. This is in accord with previous findings in Europe and the UK where perceived diversity was measured at the neighborhood level (Kouvo & Lockmer, 2013; Uslaner, 2010), as in our case, but contrary to findings where perceived diversity is assessed at the national level (Hooghe & de Vroome, 2015); this suggests that the spatial scale at which perceived ethnic diversity is measured may be important for the effects is has upon perceptions of social well-being.

There are several factors related to the regeneration context of these areas that may explain why perceived diversity is positively associated with many (though not all) outcomes. Time has passed since non-Scottish residents first arrived in these areas, and initial problems regarding conflict and harassment have been largely overcome, so that perceived diversity can now be associated with safety, particularly given residents’ knowledge of the past. It is also relevant that many residents in the regeneration areas had previously reported concerns about high levels of antisocial behavior, mainly perpetrated by White Scottish residents (Egan, Neary, Keenan, & Bond, 2013), so that in this context growing ethnic diversity may be seen as a dilution of this issue.

Further, a longer duration of experience of people can generate familiarity and friendships; a number of past studies have reported more positive effects of perceived diversity (or absence of negative effects) where interethnic friendships exist (Gorny & Torunczek-Ruiz, 2014; Uslaner, 2010), although this was not something we were able to measure in our study. A weak sense of community in regeneration areas as a result of residential instability (Bailey et al., 2012) also provides an opportunity for migrant groups, with some help from other local people, to make a contribution over time, through developing self-support and new community structures where these were absent and contributing to existing community groups in ways that develop more positive feelings about place, as found in other UK studies (Bowes, Ferguson, & Sim, 2009; Sim & Bowes, 2007; Stewart & Shaffer, 2005). In addition, regeneration areas received additional state funding and services to support the social integration and safety of migrants, and these are likely to benefit all residents (FMR Research, 2003; ODS Consulting, 2007). Given these circumstances, it is understandable that perceived diversity can be associated with positive social outcomes for residents in this context.

To a lesser extent, similar positive associations between perceived ethnic diversity and social outcomes were found in the “other” deprived areas that we studied. These are areas where ethnicity is much lower and has been increasing slowly over time; for example, increasing by four to six percentage points in each of the city’s large outer estates between the 2001 and 2011 censuses (Freeke, 2013). This lower presence and slower change has enabled these areas to avoid many negative effects of changes in ethnic composition, such as intergroup competition for housing or other local resources (Esses et al., 1998). In such a context of long-term deprived areas, perceived ethnic diversity (if allied to higher skills and aspirations) can also be associated with positive change after periods of little or no socioeconomic progress (Arnott, 2006).

Lastly, the surrounding areas constitute an unusual context. These are areas that were relatively stable residentially until regeneration nearby started to produce changes from the mid-2000s onwards. At the start of our study, whereas the regeneration areas contained 40–60% ethnic minorities, the surrounding areas were overwhelmingly White but subsequently contained a relatively high proportion of relocated in-movers from the regeneration areas, including minorities (Kearns, 2014). Thus, although ethnic diversity is much lower than in the regeneration areas, it is more recent and identifiable as a product of the relocation process. In this context, we found that perceptions of diversity had mixed associations with residential and safety outcomes and very few associations with either cohesion or empowerment outcomes. We think that these findings reflect the fact that ethnic diversity in the context of surrounding areas is a more recent phenomenon and one over which there is no local control and there is an absence of the supportive social projects that had been enacted in the regeneration areas. There may also be an effect of competition here, because the only new housing developed in these areas has been provided under a “reprovisioning” program for residents relocated from the regeneration areas, commonly asylum seekers and refugees, so that perceptions of diversity may be intimately connected to views about a lack of access to new housing for preexisting residents (Glasgow City Council, 2005).

### Strengths and limitations

Our analyses are based on a reasonably large sample of residents in three types of areas defined by regeneration in the city. Also of note is the relatively large number of ethnic minorities included in the sample, including refugees and asylum seekers, who are often underrepresented or overlooked in survey data. Previous work on perceptions of diversity in the UK have used the UK Citizenship Survey, which asks respondents to estimate what proportion of the people living in their neighborhood come from the same ethnic group as themselves. This measure of perceived ethnic density places the emphasis on homogeneity, not difference and responses, will reflect how people value sameness. In contrast, our question about perceived ethnic mix encourages respondents to directly evaluate diversity, making it potentially more pertinent. In addition, we have extensive information on neighborhood outcomes of particular relevance in this context. Our survey focuses upon the area within a 10-min walk of respondents’ homes. This has much greater salience in terms of people’s experience of ethnic diversity in their neighborhood than objective measures of ethnic diversity constructed from the UK census for much larger areas with an average population of over 7,000 residents. Indeed, researchers who have used census measures at the middle super output area level have acknowledged this lack of correspondence to actual neighborhoods as a weakness of previous work (Stafford et al., 2009; Twigg et al., 2010). However, there are also a number of limitations to consider.

Our data are cross-sectional and we are therefore not strictly able to establish whether perceived ethnic diversity is a predictor of neighborhood outcomes or vice versa, although, in practice, the two are likely to be interconnected. The other main limitation to our study of diversity is that we do not have objective data on actual ethnic densities for our study areas to consider alongside perceived diversity and we are not able to examine the effects of different kinds of diversity. We are also unable to examine how the effects of perceived diversity might be moderated by interethnic social ties.

## Conclusion

We have studied associations between perceived neighborhood ethnic diversity and social and psychosocial outcomes for residents in deprived areas in the context of a postindustrial city, Glasgow, that has a relatively low ethnic minority population but a rapidly rising presence in some neighborhoods as a result of European migration and asylum seeker dispersal and settlement over the past decade. This is an important setting given that perceived diversity has tended to be studied in cities with longer traditions of diversity and larger ethnic minority populations, though there is interest and concern in how other (often disadvantaged, second- or third-tier) cities are coping with the effects of “new migration” (Robinson & Walshaw, 2012).

Our study is unusual in looking at perceived diversity at the local neighborhood scale, rather than at the spatial scale of a town, city, or nation; this is the scale at which migrants have reported feeling the process of integration working in Scotland (Mulvey, 2013). In doing this, we have been able to show that the effects of perceived diversity are context dependent at an intra-urban scale, even within a predominantly deprived city. Moreover, the different residential contexts within which diversity exists and is perceived are affected by regeneration processes, which are common in postindustrial cities in Europe and the UK (Gospodini, 2009; Governa, Rossignolo, & Silvia, 2009). The different stages and locations of regeneration result in a variety of demographic scales (numbers and concentrations) and temporal experiences of diversity. In the most deprived areas, where regeneration is enacted first and foremost and numbers of migrants are relatively high, earlier negative associations with perceived diversity can be reversed over time as migrants are supported to develop structures that fill gaps in the local community. In adjacent areas affected by relocation, a more recent growth in diversity in smaller numbers can result in mixed associations with positive and negative outcomes. In other deprived areas, further away from regeneration activity, a slower growth in diversity with smaller numbers produces some positive associations with social outcomes but also a negative association with area reputations that are difficult to shift without substantial changes such as those produced by regeneration (Kearns, Kearns, & Lawson, 2013).

In sum, our study shows that the associations of perceived diversity with residential, social, and psychosocial outcomes are nuanced and context-dependent *within* cities. Moreover, in deprived cities, processes of regeneration serve to alter residential contexts both directly and indirectly, thus enabling factors of scale and time to mediate the relationship between perceived diversity and other outcomes in different ways in different places.

## Note

1. We have focused on psychosocial and social outcomes relating to the neighborhoods and communities in which people live, but it is recognized that regeneration also seeks to achieve physical and economic outcomes that are not considered here.
